# Lower Urinary Tract Disorders as Adverse Drug Reactions—A Literature Review

**DOI:** 10.3390/ph16071031

**Published:** 2023-07-20

**Authors:** Lukasz Dobrek

**Affiliations:** Department of Clinical Pharmacology, Wroclaw Medical University, 50-556 Wroclaw, Poland; lukasz.dobrek@umw.edu.pl

**Keywords:** adverse drug reactions, lower urinary tract symptoms, urinary retention, urinary incontinence, urinary tract infections, urolithiasis, erectile dysfunction, retroperitoneal fibrosis

## Abstract

A potential complication of pharmacotherapy for a given patient is the possibility of various side effects of drugs, which are manifested in many ways and constitute iatrogenic causes of diseases. Among the systemic side effects of drugs, there are also those involving the urinary tract, although these are less reported in the literature. The use of numerous drugs—especially of anticholinergics or drugs with anticholinergic potential, opioid analgesics, non-steroidal anti-inflammatory drugs, antidepressants, first-generation antipsychotics (classic neuroleptics) and selected cardiovascular drugs (beta-blockers, thiazides potassium-sparing diuretics, statins), as well as others—may increase the risk of developing urological disorders, such as urinary retention or incontinence, urinary tract infections, urolithiasis, erectile dysfunction in men and retroperitoneal fibrosis. The purpose of this paper is to characterise the abovementioned drug-induced disorders of the lower urinary tract on the basis of a non-systematic literature review.

## 1. Introduction

The primary goal of pharmacotherapy is to be effective and safe. Attaining this goal is related to demonstrating an acceptable risk–benefit ratio of the drugs that are currently on the pharmaceutical market. However, this fact does not mean that the use of drugs is immune to side effects that can be manifested in various systems and organs, including the kidneys and urinary tract. Therefore, lower urinary tract disorders may be drug-induced disturbances.

The term “side effect of the drug”, also known as “adverse drug reaction” is defined in different ways. The World Health Organization (WHO) defines an adverse drug reaction (ADR) as “a response to a medicine which is noxious and unintended, and which occurs at doses normally used in man” [1]. ADR was defined by the Food and Drug Administration as “any adverse event (untoward medical occurrence) caused by a drug” [2]. Moreover, the European Medicines Agency provided a definition of ADR, given in Article 1 of Directive 2001/83/EC, that is similar to the WHO’s definition: “a response to a medicinal product which is noxious and unintended.” The well-regarded Council for International Organizations of Medical Sciences (CIOMS), in its explanation of the term ADR in its recently published dictionary, provides the following definition of an ADR: “a response to a medicinal product which is noxious and unintended” [3]. Their explanation clarifies that a “response in this context means that a causal relationship between a medicinal product and an adverse event is at least a reasonable possibility. Adverse reactions may arise from the use of the product within or outside the terms of the marketing authorisation or from occupational exposure. Conditions of use outside the marketing authorisation include off-label use, overdose, misuse, abuse and medication errors.” An ADR is also known as a side effect [4].

Another commonly accepted definition of an ADR is that of Ivor Ralph Edwards: “an appreciably harmful or unpleasant reaction, caused by an intervention related to the use of a medicinal product, which predicts hazard from future administration and warrants prevention or specific treatment, or the alteration of the dosage regimen, or the withdrawal of the product” [5]. A similar characterisation of an ADR was given by Schatz and Weber [6]: “an unwanted, undesirable effect of a medication that occurs during usual clinical use.” Thus, researchers define the concept of an adverse drug reaction differently, and various definitions highlight different aspects of this phenomenon. However, the common denominator of all definitions of an ADR is the direct causal relationship between the use of the medicine and the disorder present. The causes and nature of adverse drug events are often complex and multifactorial. A detailed classification of adverse reactions in terms of their causes and mechanisms, as well as a description of factors that are predisposing to their occurrence and general rules for the collecting and reporting of ADRs, is outside the scope of this review. However, this information can be found in the narrative papers in this review [5,7,8,9,10].

The occurrence of ADRs is a significant clinical problem because ADRs are considered a cause of unscheduled hospital admissions that may persist after hospital discharge. Between 5% and 10% of patients are estimated to suffer from an ADR at admission, during admission or upon discharge, despite various preventative efforts [10]. Adverse reactions are more common in older patients, with a higher pathophysiological burden at the baseline and in those undergoing polypharmacotherapy. The prevalence of ADRs in older adults is approximately 11.0%. ADRs leading to urgent hospitalisation is 3.3% [11]. In summary, ADRs are regarded as the most common iatrogenic illness, complicating 5 to 15 per cent of therapeutic drug courses [12].

The classes of drugs that are regarded as being associated with the highest occurrence of ADRs include agents used in the treatment of cardiovascular disorders (beta-adrenergic blocking agents, diuretics, angiotensin converting enzyme inhibitors); antiplatelets; warfarin and other anticoagulants; antipsychotic agents; non-opioid and opioids analgesics; cytotoxics; immunosuppressants; antidiabetics; and antibiotics [10,13].

Since many drugs are administered via the *per os* route and pass through the digestive tract, gastrointestinal disturbances (loss of appetite, nausea, abdominal pain, bloating sensation, constipation, and diarrhoea) are common types of ADRs. Various dermatological disorders are also frequent adverse drug reactions [11]. However, in principle, adverse drug reactions can manifest in all systems and organs. The report by CIOMS distinguishes 21 anatomical and physiological areas for ADR reporting purposes and includes drug-induced disturbances of the urinary system [14].

Among the drug-related disturbances of the urinary system (SOC1300) distinguished in this report, 11 were distinguished as predominantly affecting the kidneys and urinary tract: glomerular vasomotor disorder; glomerulonephritis (acute or chronic); nephritis interstitial (acute or chronic); nephropathy analgesic; nephropathy toxic; nephrotic syndrome; kidney failure; acute kidney failure; kidney tubular disorder; kidney vasculitis; and urinary retention (UR).

Some of these disturbances are potentially severe (e.g., acute tubular necrosis and glomerulonephritis), or affect the kidney in a significant way as part of a systemic disorder (e.g., vasculitis). The main mechanisms by which drugs cause kidney dysfunction include pre-kidney effects (e.g., water or electrolyte loss, increased catabolism, vascular occlusion or altered kidney haemodynamics); obstructive uropathy (due to tubular blockage, ureteric fibrosis or calculi); allergic or immunological damage (resulting in vasculitis, interstitial nephritis or glomerulonephritis); and direct nephrotoxicity (giving rise to acute tubular or interstitial damage or kidney papillary necrosis) [14].

The list of drug-induced urinary tract disorders mentioned above does not include many lower urinary tract disorders other than UR, although the report mentions that drugs can also have adverse effects on the bladder or urothelium, such as haemorrhagic cystitis or carcinoma of the urinary tract.

The PubMed database searches, performed in June 2023 and limited to English-language records from the last 10 years, used the search terms “adverse drug reactions” and “lower urinary tract” or “urological” yielded 170 and 576 results, respectively. At the same time, when searching the PubMed database for records relating to dermatological or gastroenterological adverse drug reactions, a much larger number of publications was found (for the search terms “adverse drug reactions” and “dermatological”—2901 items, and for the entries “adverse drug reactions” and “gastrointestinal”—2982 items). Thus, the performed query indicates that drug-induced urological disorders are less known and less reported in the literature.

Therefore, the aim of the review was to discuss the most recognised, in the author’s opinion, drug-induced, adverse lower urinary tract disturbances described in the literature: urinary retention or incontinence, urinary tract infections, urolithiasis, erectile dysfunction and retroperitoneal fibrosis. The intention of the author was to organise and collect information on the above-mentioned urological disorders, discussing their potential drug-induced causes. This paper is based on the literature references covering adverse drug reactions in the form of lower urinary tract disturbances found through Medline/PubMed searches. In addition, the query was supplemented with references to key urological and pharmacological texts mentioned in the articles originally obtained during the literature review. It should also be emphasised that the drug-induced background of the urological disturbances described in this review is one of the elements of the broadly understood differential diagnosis of these disorders. Urological ADRs, like any other ADRs, are difficult to identify, and their diagnosis is based on the exclusion of other, organic or functional potential causes of the observed disorders, as well as on the demonstration of the existence of a cause-and-effect relationship between the observed disorder and the use of a given drug. It is also important to demonstrate the resolution or recurrence of the diagnosed urological disorders in the case of discontinuation of treatment, as well as during re-exposure to the drug used, respectively. Thus, this review describes the potential mechanisms predisposing to the development of urological ADR, which does not mean that in each case of using a specific drug the observed disorders are undoubtedly drug-induced.

## 2. Urinary Retention or Urinary Incontinence

The most important function of the kidneys and urinary tract is the excretion of urine containing metabolic wastes and drugs with their metabolites, which is one of the vital functions of the human body. The voiding process is under the control of complex peripheral neural pathways that are, in turn, coordinated by cell groups in the spinal cord, brainstem and brain. There are two complementary stages: the storage of urine inside the bladder (“filling”) and (2) the elimination of urine through micturition, involving bladder emptying and urinary outlet (it consists of the bladder neck, the urethra and the urethral sphincters) [15]. The storage reflex maintaining continence is based on the bladder distention that enables bladder filling. The bladder normally accommodates up to 300–400 mL in adults. Moreover, the filling phase is characterised by the voluntary contraction of the external urethral sphincter, with the sympathetic contraction of the inner urethral sphincter [16].

During bladder filling, the distention of the smooth muscle and urothelium evoke afferent activity. The afferents from stretch and volume receptors located at myelinated Aδ sensory fibres are conveyed via the pelvic (parasympathetic nervous system) and hypogastric nerves (sympathetic nervous system), whereas the pudendal (somatic nervous system) and hypogastric nerves carry impulses from the neck of the bladder and the urethra. Moreover, in some conditions such as overactive bladder or urinary tract infections, afferent activity also involves unmyelinated C-sensory fibres, which physiologically have a high mechanical threshold but respond to many neurotransmitters and other inflammatory mediators. These neuromodulators lower the threshold for bladder contraction and provoke symptoms such as urgency, an increased frequency of urination and urinary incontinence (UI). The afferent signals are carried to the spinal cord and then via the spinothalamic tract to the periaqueductal gray (PAG) matter and pontine micturition centre (PMC; Barrington’s nucleus), which is located in the medial dorsal pons, close to, or includes the lateral dorsal tegmental nucleus and locus coeruleus. The role of the PAG in the control of the bladder function encompasses both downstream connections, as well as connections with the higher brain centres involved in decision-making and initiation of urination [17,18].

**Figure 1:**
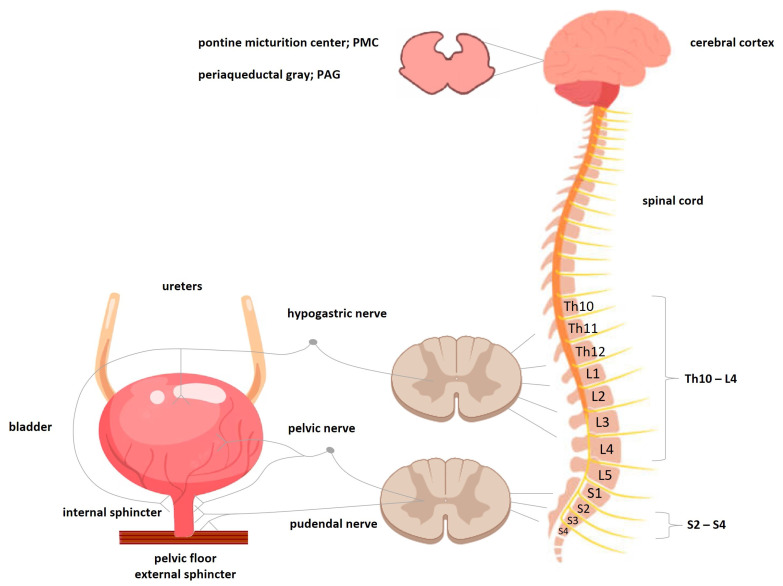
The neurological control of the lower urinary tract. The lower urinary tract receives both autonomic and somatic innervation. The hypogastric nerve (sympathetic), by releasing noradrenaline, acting via beta-3-adrenoreceptors, inhibits detrusor activity during the filling stage of the bladder and contracts muscles in the urethra and bladder neck via alpha-1-adrenoreceptors. Pelvic nerve (parasympathetic) unopposed impulses result in detrusor contraction via acetylcholine, acting at muscarinic M3 receptors. The pudendal nerve (somatic) controls the voluntary function of the striated, external urethral sphincter, acting via acetylcholine and nicotinic receptors. The cerebral cortex, through the PAG, communicates with the pontine micturition centre, which, depending on the tonic influence of the cortex, “switches” between the filling/storage and voiding phases.

The higher brain centres of micturition communicate with the PMC through the PAG matter to either provoke or suppress the voiding reflex. Along with bladder distention, the afferent signal from the stretch receptors of the bladder reaches the pons, which subsequently notifies the brain. This afferent signal results in a perception of bladder fullness or the desire to urinate. The higher brain (anterior cingulate and the prefrontal cortex) determines the appropriateness of initiating the voiding reflex. Mostly, the higher brain suppresses PMC by transmitting reciprocal inhibitory signals via the PAG matter. It abolishes the urge to urinate and allows delaying voiding until finding a socially acceptable place and time. The voiding is initiated in an appropriate time and place, when the suppression of the PMC disappears, enabling a micturition reflex activation [19]. The efferent pathways involve both sympathetic and parasympathetic fibres, as well as somatic ones. Sympathetic fibres originating from the T11-L2 spinal segments are part of the hypogastric nerve and link to the base of the bladder and urethra. In the filling period of the bladder, noradrenaline released from the postganglionic sympathetic terminals acts on the beta-3 adrenergic receptors in the bladder, which contributes to the relaxation of the detrusor muscle, and on the alpha-1 adrenergic receptors in the proximal urethra, causing its contraction. The parasympathetic preganglionic fibres originate from the S2–S4 spinal segments and they travel in the pelvic nerves and link to the bladder wall. During bladder emptying, an increased parasympathetic efferent drive occurs, which is associated with the release of acetylcholine from parasympathetic terminals. As a consequence, the activation of muscarinic receptors M1-M3 occurs. The predominant muscarinic receptor is the M3 one, and the activation of this receptor results in bladder contraction. M1 receptors appear to facilitate the further release of acetylcholine, while the activation of M2 receptors leads to the inhibition of detrusor relaxation by diminishing sympathetic activity. This enhances the detrusor response to M3 receptor activation. Also, somatic motor nerves from the S2–S4 motor neurones travel in the pudendal nerves and link to the striated muscles of the external urethral sphincter via nicotinic receptors, enhancing its contraction. The external sphincter consists of striated muscle and is similar to the skeletal muscle under voluntary control [16,18,20]. The organisation of the micturition reflex is shown in Figure 1.

Thus, it can be concluded that the expulsion of urine requires the proper coordination of the contraction and relaxation of the bladder and bladder outlet, respectively, coordinated by the CNS. Both UR and incontinence belong to the “lower urinary tract symptoms; LUTS”, a term that encompasses all urinary symptoms, namely, voiding, storage, and post-voiding [21,22]. LUTS has replaced the previously used terms “failure to store” and “failure to empty”.

The inability to generate efficient bladder emptying leads to UR. It is a clinical condition that is classified as acute or chronic. Acute UR is characterised by a rapid onset associated with suprapubic pain and the inability to urinate. Conversely, chronic UR is not associated with pain due to the fact that small amounts of urine still may be exposed from the body. Paradoxically, patients with chronic UR can present symptoms of UI, occurring after exceeding the critical amount of urine and intravesical pressure. The incidence of UR is higher in men compared to women and shows an increasing trend with age [23]. UR may result from various disturbances, including the most common bladder outflow obstruction (at the level of the bladder, e.g., calculi, blood clot, tumour; the prostate, e.g., benign prostatic hyperplasia, calculi, prostate carcinoma; urethra, e.g., stones, strictures, diverticulum, posterior urethral valves, surgery). The other etiological factors of UR include acute pain, neurogenic factors (“neurogenic bladder”) in the course of focal lesions (stroke, tumour, traumatic spinal cord injury); disseminated lesions (Parkinson’s disease, brain trauma, multiple sclerosis); peripheral neuropathies (diabetes mellitus); and idiopathic myogenic factors affecting the contractile activity of the myocytes (e.g., disruption of ion storage/exchange, excitation-contraction coupling, calcium storage and energy generation). Recumbency and post-operative conditions are also common causes of UR in clinical practice [23,24,25]. At this point, it should also be mentioned that a number of terms have been proposed to define a dysfunctional bladder: detrusor areflexia, hypotonic bladder, detrusor failure, and chronic retention. To characterise chronic UR, the term “underactive bladder; UAB”, characterised by detrusor underactivity (DU) and analogous to overactive bladder (OAB) and detrusor overactivity, has also been introduced [24]. According to the International Continence Society, UAB has also been defined as a complex symptom suggesting DU, characterised by prolonged urination time with or without a sensation of incomplete bladder emptying, usually with hesitancy, reduced sensation on filling and a slow stream, while DU is defined as a contraction of reduced strength and/or duration, resulting in prolonged bladder emptying within a normal time span [26].

**Figure 2:**
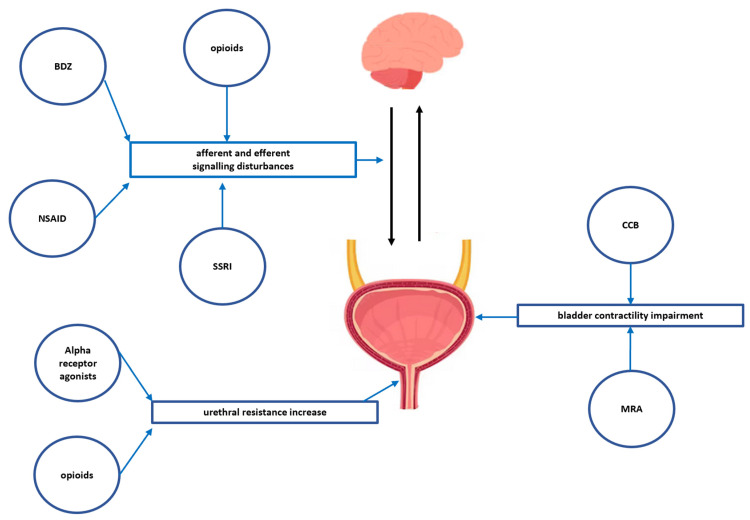
The main mechanisms contributing to potential drug-induced urinary retention (BDZ–benzodiazepines; NSAID–non-steroidal anti-inflammatory drugs; SSRI–selective serotonin reuptake inhibitors; CCB–calcium channel blockers, MRA–muscarinic receptor antagonists (anticholinergics).

Considering the complex mechanism of voiding outlined above, it is possible to identify classes of drugs which can interfere with this process, resulting in either UR or UI. The mutual afferent and efferent signalling between the bladder and urethra and the CNS, contributing to urinary retention, is disturbed by opioid drugs (by affecting opioid receptors); non-opioid analgesics (by affecting tachykinin receptors); benzodiazepines (by affecting GABA receptors); and serotonin reuptake inhibitors (by potentiating serotonin-dependent increased sympathetic activity that increases bladder compliance). On the other hand, drugs with the ability to antagonise muscarinic receptors (anticholinergics) impair the cholinergic mediated contractility of the bladder. Similarly, bladder contractility is impaired by calcium channel blockers through a direct effect on smooth muscle through the inhibition of calcium channels. Drug-induced urinary retention may also result from the stimulation of alpha-1 adrenergic receptors, which leads to contraction of the internal urethral sphincter and impairment of urinary outflow. A schematic representation of the above mechanisms contributing to potential drug-induced urinary retention is shown in Figure 2.

Taking into account the autonomic control of urination, it can be concluded that drugs affecting the mutual communication between the CNS and the lower urinary tract, those inhibiting the parasympathetic system (muscarinic M3 receptor antagonists) as well as those stimulating the sympathetic system (alpha-1 receptor agonists) may disturb the micturition process, contributing to drug-induced UR [27,28]. It should also be underlined that most of the aforementioned drugs are used for the treatment of the opposite disorder to UR–bladder overactivity. The drugs with an increased risk of causing UR, with detailed mechanisms contributing to this disorder, are listed in Table 1.

The opposite disorder to UR is UI. It is a condition characterised by the involuntary loss of urine. UI can be diagnosed in childhood and may resolve itself with growth; however, it usually occurs in patients over 40 years of age and increases with ageing [29]. UI is regarded as affecting mostly women. The estimates indicate that the prevalence of UI is about 19% in women younger than 45 and reaches about 29% in women 80 years or older [29,30]. Although UI often affects women, a significant number of males also suffer from UI. It is reported that 12–17% of males are affected by UI, and the incidence rate increases with age. Besides age, the other risk factors include obesity, parity, smoking, diabetes and hysterectomy [29,31]. Both acute, often transient UI and chronic UI can be distinguished. Transient UI develops suddenly, lasts less than six months and is reversed as the underlying disease subsides. Based on the pathomechanism, four main types of incontinence may be distinguished: stress, urge, mixed and the overflow form. The stress UI is observed mostly in women and results from weakened bladder sphincteric mechanisms that are not able to fully protect against a leakage of urine. This kind of UI is observed during coughing, sneezing or other phenomena associated with increased abdominal pressure. The increased abdominal pressure exceeds the intravesical pressure, and in the case of an insufficiency of the bladder outlet and sphincter resistance, an uncontrolled leakage of urine occurs. Urge UI affects both genders and is caused by the sensation of a sudden desire to void that cannot be postponed due to the involuntary detrusor contractions during the filling of the bladder. Mixed UI is a combination of both the aforementioned mechanisms. Overflow UI affects mostly men and is characterised by the overfilling of the bladder that could not be emptied [29,32,33]. There is also an idiopathic, primary OAB that is a functional disease of the lower urinary tract. As also mentioned above, this clinical entity is characterised by urinary urgency with or without UI and with nocturia and the increased daily frequency of voiding episodes. If there is no proven, obvious pathology, there are some pathomechanisms proposed to explain the OAB development, including primary, myogenic detrusor overactivity (revealed in urodynamic recordings); dysfunction of the afferent arm of the micturition reflex (with lowering the threshold of the excitation of afferent fibres); and a urothelial dysfunction or dysfunction at the level of the higher central nervous system (CNS) responsible for the perception of the bladder filling phase [20,34].

**Figure 3:**
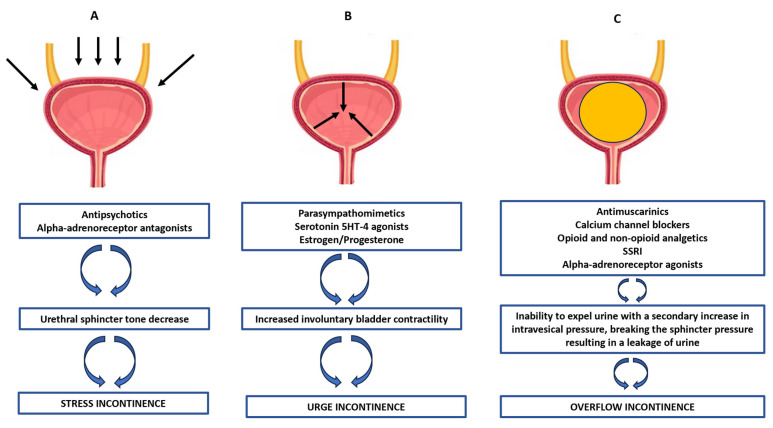
The main types of urinary incontinence based on the prevailing pathomechanism, along with listing the most important classes of drugs contributing to the development of urinary incontinence (SSRI–selective serotonin reuptake inhibitors). The arrows in panel (**A**) show the increase in intra-abdominal pressure; the arrows on panel (**B**) show the spontaneous increase in bladder contractile activity; panel (**C**) shows the bladder overfilling with urine.

UI can be an isolated problem, or it can be the result of an underlying disease affecting the nervous system and the autonomic control of urination, such as stroke, myelomeningoceles, Parkinson’s disease, Alzheimer’s disease, multiple sclerosis or neuropathies. Moreover, there are some specific causes of male UI, including benign prostatic hyperplasia, prostate cancer and related surgery or radiation therapies. These types of UI are collectively named incontinence after prostate treatment (IPT) [31]. In the differential diagnosis of UI, it should also be taken into account that UI may also be a consequence of the administration of some drugs. The basic mechanism of drug-induced UI is related to the impairment of the bladder storage phase by the lowering of the bladder outlet resistance and/or by increasing intravesical pressure. Under physiological conditions, during filling, the intrabladder filling pressure is still lower than the resistance of the sphincter and urethra. Thus, competent bladder compliance and sphincter mechanisms are of key importance in the period between micturitions; and any defect affecting this system may result in the involuntary loss of urine [27,32]. A variety of drugs have been directly implicated in disturbances of the storage phase by the general weakening of the sphincter tone (contributing to stress UI), e.g., adrenergic alpha receptor antagonists or antipsychotics (via complex action on the central and peripheral adrenergic, muscarinic, serotonin or GABA receptors). There are also drugs evoking uninhibitable bladder contraction activity (leading to urge UI), resulting from direct stimulation of muscarinic receptors in the bladder or complex CNS action. UI may be also conditioned indirectly, by diminishing bladder emptying leading to bladder overdistention (overflow UI). Thus, the drugs mentioned above that are potential causes of UR may also consequently cause UI. The main mechanisms contributing to UI with an indication of the main classes of drugs in a given type of urinary incontinence are presented in Figure 3.

According to the pathophysiological premises described above, numerous drug groups can theoretically induce UI. However, drug-induced UI has been well evidenced for only a few classes (listed in Table 2), and the relationship between the administration of many drugs and UI still remains sparse and uncertain. Moreover, it should also be noted that the drugs causing UR discussed in the previous paragraph may also thereby cause overflow incontinence [32,33,35]. Moreover, it is worth noting that the overproduction of urine caused by diuretics may lead to UI. The drugs with an increased risk of causing UI are listed in Table 2.

## 3. Urinary Tract Infections

**Figure 4:**
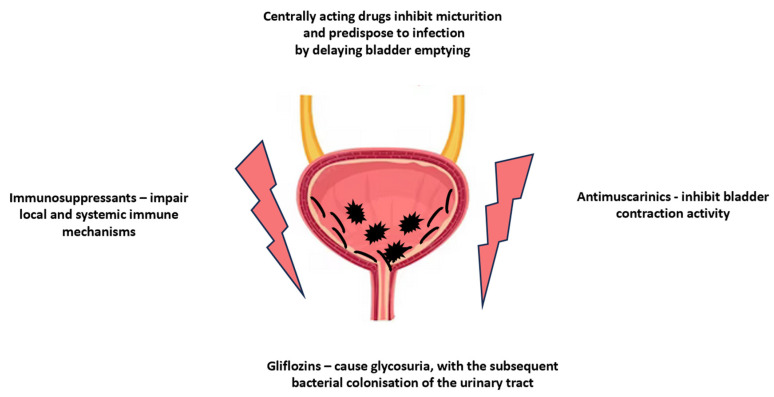
The main drug-related mechanisms contributing to urinary tract infection development. “Lightning bolts” indicates the development of inflammation; uropathogens colonizing the bladder are marked in black.

An uncomplicated urinary tract infection (UTI) is an infection of the lower urinary tract (urethra and bladder), developing in patients without structural abnormality and comorbidities, such as diabetes, immunocompromised state, pregnancy, kidney transplants, the presence of a urinary catheter or other factors predisposing them to a complicated course of the infection. It is commonly known as cystitis or lower UTI, and it manifests itself with typical symptoms including urinary frequency, urgency, suprapubic discomfort and dysuria. UTI is usually caused by enteric coliforms that typically inhabit the periurethral vaginal introitus, with Escherichia coli as the main etiological factor constituting the vast majority of UTIs, followed by Klebsiella, and other organisms (Proteus, Enterobacter, and Enterococcus). Uncomplicated UTI involves the bladder starting with the invasion of the bladder mucosal wall by bacteria using adhesins on their surface, which allow them to attach to the urothelial mucosal surface. In addition, a short urethra makes it easier for the uropathogen to invade the urinary tract; thus, UTIs are significantly more common in women. Estimates indicate that 40% to 60% of women will have a UTI episode at least once in their lives, which means that UTIs are observed four times more frequently in females than males. A UTI may also progress to further complications: increasing the risk of urinary calculi development, UI, chronic prostatitis and affecting the upper urinary tract (pyelonephritis, kidney abscess, kidney failure) [36,37]. The pathophysiology of infectious UTI is beyond the scope of this review and can be found in some papers in this field [38,39,40]. However, UTI development may also result from the patient’s pharmacotherapy. Thus, urinary tract infections may be a urological manifestation of adverse drug reactions. One of the classes of drugs with the best-documented effect on the development of UTI are gliflozins (inhibitors of kidney sodium-glucose co-transporters SGLT2), which directly results from their mechanism of inhibiting the reabsorption of glucose from the urine. As a consequence, glycosuria occurs, which is the basis for the urogenic, ascending urinary and genital tract infections [41]. However, there are also other drugs that predispose to the development of UTIs. The other main mechanisms accounting for a drug-induced UTI include immunodeficiency in the lower urinary tract (as a result of systemically or topically acting immunosuppressive and cytotoxic drugs); the impairing of micturition and bladder emptying (due to the cholinolytic activity or the increasing of the bladder outlet resistance evoked by the drugs mentioned above); and impairment of the neurogenic control of the bladder. They are summarised in Figure 4.

Moreover, the complementary mechanism contributing to UTI is urine stagnation and retention secondary to urinary stone formation in the urinary tract, as well as the intensification and promotion of the bacterial colonisation of urine (as a result of drugs that intensify glycosuria) [41]. The drugs with the potential to cause UTIs are listed in Table 3.

Moreover, cystitis may be non-infectious and occur in the form of severe, hemorrhagic damage to the bladder. Thus, drugs that cause the development of the so-called chemical cystitis (CC) and its complication–hemorrhagic cystitis (HC)–deserve special attention. CC is caused by different chemical agents, including chemotherapeutic or immunologic drugs, such as cyclophosphamide or ifosfamide (oxazophosphorine compounds), mitomycin C, anaesthetic agent–ketamine, tiaprofenic acid, danazol or agents administered intravesically (the intravesical instillation with bacillus Calmette-Guerin; BCG as prophylactic immunotherapy for intermediate or high-risk non-muscle-invasive bladder cancer, after transurethral resection of bladder tumours or diluted gentian violet is used to assess bladder injury after herniorrhaphy). CC/HC shares similar symptoms with other types of cystitis, presenting suprapubic pain, dysuria, urinary frequency, urinary urgency, and hematuria (microscopic or macroscopic). The pathogenesis of cyclophosphamide and ifosfamide-induced HC is associated with the corrosive liver product called acrolein, which is a by-product formed during the metabolism of oxazaphosphorines. Acrolein is filtered by the kidneys and consequently accumulates in the bladder where it induces a complex reaction with pyroptotic effects in the urothelium, resulting in ulceration and the exposure of the muscularis mucosa and the blood vessels. Moreover, acrolein can break down proteins and damages DNA structure, which results in a metabolite that causes apoptosis. An important element of the pathophysiology of HC caused by oxazaphosphorines is also the intensification of oxidative stress and the overproduction of highly reactive oxygen and nitrogen-free radicals [42]. A detailed description of the pathogenesis of oxazaphosphine-induced HC can be found in some of the reviews on this issue [43,44,45,46]. Ketamine (an N-Methyl-D-aspartate receptor antagonist) is a dissociative anaesthetic and psychotomimetic agent. Chronic abuse of ketamine can lead to significant ketamine-induced cystitis (KIC) that manifests with LUTS, including urinary frequency, urgency, and severe bladder pain. As the disease progresses, a contracted bladder, bladder wall thickening, petechial haemorrhage of the bladder mucosa, and ureteral stricture with hydronephrosis develop. A common finding is ulcerative cystitis with an easily bleeding mucosa. The pathophysiology of KIC is not fully understood, however, several pathomechanisms are postulated, including the direct toxic effect of ketamine, the activation of inflammatory cells, dysfunction of bladder-urothelial barrier, the dysregulation of neurotransmission, overexpression of carcinogenic genes, cell apoptosis, and oxidative stress [47,48,49].

## 4. Urolithiasis

Urolithiasis (nephrolithiasis; kidney stone disease) is a relatively common clinical entity and research indicates that the general prevalence of the disease varies between 1–5% in Asia, 5–9% in Europe, 13% in the USA to even 20% in Middle Eastern countries [50,51]. According to general aetiology, one can distinguish infectious and non-infectious stones: those caused by genetic defects and those induced as a result of adverse drug reactions. In general, urinary stone formation results from three main disturbances: (1) an excessive urinary concentration of some compounds, exceeding their solubility in the urine, (2) the presence of promoters with a simultaneous deficiency of inhibitors of precipitation and (3) urothelial abnormalities allowing an attachment and subsequent growth of rising crystals [51]. The pathophysiology of urolithiasis is complex and divided into the nucleation phase, with the subsequent crystal growth. In the second step, the microcrystals continue the overgrowth accomplished through the aggregation of the preformed crystals or secondary nucleation of the crystal on the matrix. The final step is the association and fixation of the crystals in the kidney tubule cell lining. The symptomatology of urolithiasis is dependent mainly on the size and location of the urinary stones and the presence of a possible associated urinary tract infection. Stones smaller than 5 mm are likely to pass unimpeded through the urinary tract. During the passage of the stone through the urinary tract, kidney stone symptoms present themselves. Kidney stone pain is a severe cramping pain evoked by the movement of a stone through the urinary tract, which is augmented by the ureteral spasm and possible obstruction. The pain is usually not related to body position and is accompanied by nausea, vomiting and macro- or at least micro-hematuria and often bladder overactivity symptoms (sensation of urinary frequency and urgency) [52]. The detailed description of the pathophysiology and symptomatology of urolithiasis is out of the scope of this paper and can be found in other reviews [51,53,54,55,56]. According to general aetiology, one can distinguish infectious and non-infectious stones, those caused by genetic defects and those induced as a result of adverse drug reactions. On the other hand, based on the above-mentioned chemical composition, five main types of stones can be classified: calcium, struvite or magnesium ammonium phosphate, uric acid or urate, cystine and rare stones (including drug-induced ones) [52,57].

**Figure 5:**
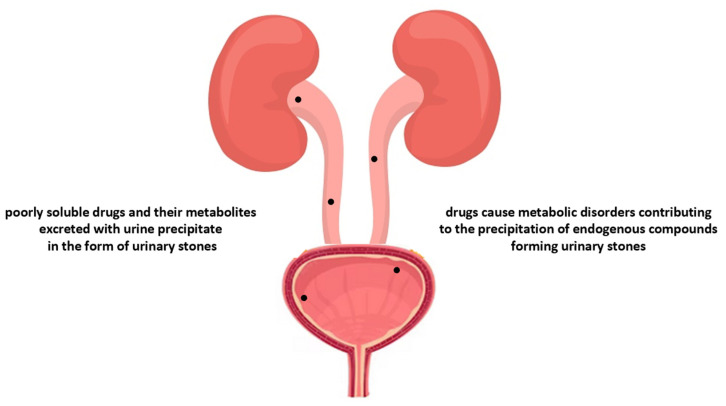
The main mechanisms responsible for the formation of drug-induced urinary stones. The dots represent urinary stones in the lower urinary tract.

Taking into account the main pathomechanism, two main types of drug-induced urinary stones can be distinguished. They are shown in Figure 5.

The first group includes stones composed principally of the drug itself and/or its metabolites excreted into the urine. They are poorly soluble compounds, for which the kidneys are the main route of elimination. Therefore, in accordance with the general pathophysiology of urolithiasis, these drugs may, in supersaturated urine, be characterised by exceeding the solubility equilibrium. The second group of drug-induced urinary stones are those classified as a sub-type of “metabolic stones”, due to the fact that treatment with these drugs contributes to the development of metabolic disturbances that facilitate the crystallisation of endogenous lithogenic substances. Many drugs may induce urinary stone development by affecting the pH of urine (in such a way that the solubility of many endogenous substances decreases), the alternation of the glomerular filtration and tubular secretion/reabsorption of the endogenous substances, or impairing the balance and action of crystallisation promoters/inhibitors [52,58,59,60]. The drugs contributing to drug-induced kidney stone development are listed in Table 4.

## 5. Erectile Dysfunction

**Figure 6:**
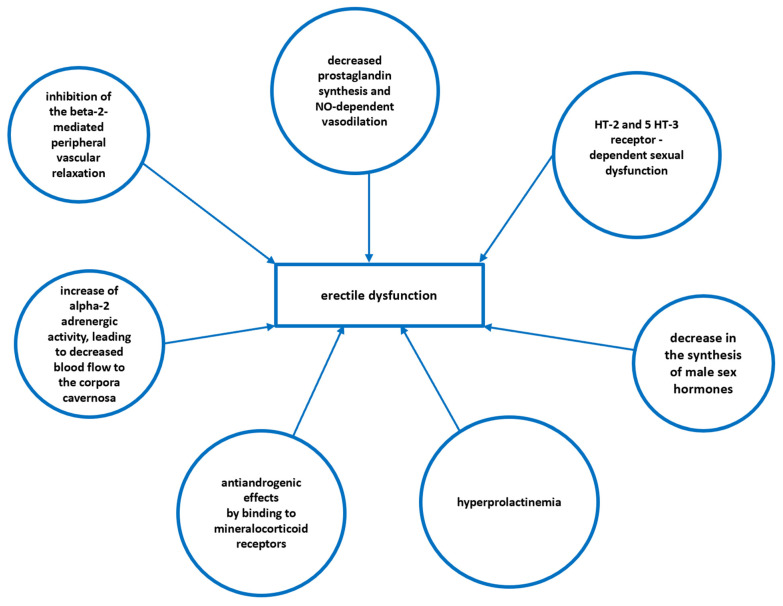
The main drug-related mechanisms contributing to erectile dysfunction.

There are many causes of erectile dysfunction (ED) development in men, including pathophysiological (hypertension, hyperlipidemia, diabetes) and psychological factors. However, there are also iatrogenic causes of ED, including drugs. Several drug-dependent mechanisms can be implicated as potentially contributing to the ED development. Among them, there is an antiandrogenic effect, hyperprolactinemia development, a decrease in the level of androgens, disorders of autonomic innervation or impaired peripheral circulation. They are shown in Figure 6.

Numerous drugs may affect sexual functioning with important consequences related to patients’ quality of life. Sexual dysfunction in men can also worsen treatment results by decreasing a patient’s adherence to a programme of medication. This is of special importance in the case of drugs which are required to be precisely administered to evoke a desired pharmacological effect, e.g., anti-HIV medications, anticonvulsants or immunosuppressants. Moreover, given the embarrassing nature of ED, the true incidence of this ADR appears to be unknown and underestimated. ED is the main reason for the premature termination of antidepressant treatment, which should normally last at least 6 months. Approximately 42% of men discontinue antidepressant treatment after the development of disturbances to their sexual desire, ejaculation and orgasm [64]. ED is also noted mostly in patients suffering from benign prostatic hyperplasia (BPH) and LUTS—estimates indicate that over 70% of such BPH patients have sexual dysfunction and treatment with 5-alpha-reductase inhibitors may exacerbate the phenomenon [65]. Other classes of drugs considered to have the greatest impact on ED include cardiovascular drugs, antidepressants, selected antipsychotics, antiepileptics, non-steroidal anti-inflammatory drugs, muscle relaxants and H2-receptor antagonists [65,66,67]. They are listed in Table 5.

## 6. Retroperitoneal Fibrosis

Retroperitoneal fibrosis (RPF) is a rare condition that is characterised by chronic inflammatory of, and fibrosis in the retroperitoneum, resulting in a midline plaque, usually at the aortic bifurcation. The gross appearance of RPF is that of a smooth, flat, tan-coloured dense mass that encases the retroperitoneal structures, usually centred at the fourth and fifth lumbar vertebrae. The extensive fibrotic process often affects the ureters and may lead to ureteric obstruction. RPF is observed in patients aged 40–60 years, with male predominance (the male-to-female ratio is estimated to be approximately 2:1 or 3:1). The total incidence of the disease is estimated to be 1 per 200,000 to 500,000 per year [68]. The clinical manifestation of RPF involves backache, abdominal pain, hydrocoele, oedema or anuria. The etiology of RPF is unclear, and in most cases (70%), an idiopathic mechanism of its development is mostly considered. In line with the assumption, an idiopathic RPF is regarded to be a manifestation of a systemic autoimmune disease, which may arise as a primary aortitis that elicits a periaortic fibro-inflammatory response. The factor suspected of triggering and sustaining the immune reaction and fibrosis is ceroid–a complex polymer of oxidized lipids and protein found in atherosclerotic plaques, with cellular infiltration including IgG4 positive plasma cells and lymphocytes. About 30% of RPF cases are the result of an identifiable cause. Malignancy (carcinoid, Hodgkin and non-Hodgkin lymphoma, sarcomas); infections (tuberculosis, histoplasmosis, actinomycosis); and radiation therapy for testicular seminoma, colon and pancreatic cancer, retroperitoneal haemorrhage and surgery have also been identified as secondary causes of retroperitoneal fibrosis. In addition to malignancy, autoimmune disorders or radiation therapy, the long-term intake of certain drugs is also a suggested risk factor of iatrogenic RPF [68,69]. The detailed pathogenesis of drug-induced RPF has been not satisfactorily described, although it might consist of a drug-haptenic role or, alternatively, it may be related to the release of phlogogenic mediators (histamine, kinins, prostaglandins) and fibrotic markers (transforming growth factor-1; TGF-1, basic fibroblast growth factor; bFGF, platelet-derived growth factor; PDGF). Finally, TGF/Smads cascade-mediated enhancement of myofibroblast proliferation with the following overproduction of extracellular matrix (ECM) components, such as collagen, fibronectin, tenascin and glycosaminoglycans, occurs [68,69,70]. The observational studies indicate that the risk of RPF developing is most documented in cases of treatment with methysergide–an ergot derivative whose use is restricted to the prevention of severe headaches as well as for other ergot derivatives used in the treatment of Parkinson’s disease, including bromocriptine, pergolide, lisuride and cabergoline [71,72,73]. The other drugs considered to be associated with RPF are listed in Table 6.

## 7. Clinical Examples of Drug-Induced Urological Disorders

A review of clinical reports on adverse drug reactions manifesting in the lower urinary tract suggests that they are most often observed in clinical practice after treatment with centrally acting drugs. A systematic review using a meta-analysis by Trinchieri et al. [74], analysing urological disorders caused by antidepressants and antipsychotics, indicates that treatment with antidepressants is associated with an increased risk of micturition disorders (both storage and emptying disturbances). The higher odds for voiding dysfunction were found for tricyclic antidepressants and for serotonin and norepinephrine reuptake inhibitors (SNRIs). In the case of SSRI, the effect was less potent. In the analysis of antipsychotics, different types of urinary disorders including urinary retention and incontinence were reported, mostly for conventional antipsychotics, such as phenothiazines and thioxanthenes (chlorprothixene), or of some atypical antipsychotics, such as clonazine. Similar conclusions can be drawn based on the analysis of Winkler et al. [75] on urological disorders assessed in hospitalised patients receiving psychotropic drugs. Among a total population of 462,661 inpatients treated with psychotropic drugs in 99 psychiatric hospitals between 1993 and 2016, UR (129 cases, 0.028%) was the most common drug-induced LUTS, followed by incontinence (23 cases, 0.005%). A detailed analysis showed that the antidepressants with the highest risk of developing UR were amitriptyline, clomipramine, paroxetine and trimipramine. To a lesser extent, these disorders have been reported in patients treated with venlafaxine, duloxetine, mirtazapine, doxepin, escitalopram, sertraline, citalopram and trazodone. In the case of antipsychotics, UR was more common in patients treated with promethazine, haloperidol, pipamperone, olanzapine, perazine, levomepromazine, flupentixol and chlorprothixene.

For sertraline, the literature review also revealed case reports of patients with sertraline-induced both UR [76] and UI [77,78]. The risk of urological disorders increases in elderly patients, in particular those treated with antidepressants and burdened with other diseases. A case of an 86-year-old patient with benign prostatic hyperplasia was described, in whom urinary retention was observed under the influence of amitriptyline (at a dose of 10 mg daily), and withdrawal of the drug alleviated the severity of symptoms [79]. Acute urinary retention was also described in a patient with postpartum depression, treated with TCAs, who additionally was taking an antimuscarinic spasmolytic drug for dysmenorrhea [80].

The literature review also revealed a case of a 62-year-old patient with a past medical history notable for bipolar I disorder, nephrogenic diabetes insipidus, and metastatic colorectal cancer presenting with a chief complaint of non-radiating suprapubic abdominal pain. This patient was initially treated with lithium, but due to the development of nephrogenic insipidus, the therapy was changed to lamotrigine, and three weeks before the onset of the UR episode, to olanzapine. In the course of the differential diagnosis process, it was concluded that the cause of UR observed in the patient was the antimuscarinic effect of olanzapine associated with impaired micturition [81]. Olanzapine treatment was also the cause of chronic UR in a 51-year-old patient who received 22.5 mg of the drug for four years, accompanied by worsening psychotic symptoms, difficulty urinating, and blurred vision [82].

Opioids are a group of drugs whose use is also associated with an increased risk of UR in real clinical practice. In a prospective study, Panicker et al. [83] showed that in 13/61 patients treated with opioids (morphine, tramadol or oxycodone), due to the variety of pain syndromes (most often abdominopelvic pain or musculoskeletal pains or back pain due to a mechanical derangement of the spine), UR episodes were observed. Urinary retention is also associated with buprenorphine, particularly with epidural/intrathecal delivery. The buprenorphine-related acute UR was demonstrated in a 49-year-old patient with a history of opiate dependence, alcohol dependence, bipolar disorder and borderline personality disorder. He was treated with buprenorphine (8–12 mg daily) for opioid dependency. On the third day after the initiation of treatment, the patients complained about their inability to urinate with suprapubic discomfort and presented to the emergency department (ED), where 800 mL of urine was drained [84].

Benzodiazepines are the next class of centrally acting drugs whose use is also associated with voiding disturbances. In a prospective study by Landi et al., it was demonstrated that 475 individuals (21% of patients aged 60–74 years and 38% aged > 75 years) currently on a regimen of benzodiazepines presented with UI. The estimated global risk of UI during treatment with benzodiazepines was nearly 45%, and the effect was mainly associated with agents with a long half-life elimination [85]. On the other hand, benzodiazepine administration may also lead to UR development. The case of a 67-year-old male patient with alprazolam-induced UR was reported: The patient underwent a prostatectomy operation for BPH and was also treated with alprazolam 1 mg/day due to insomnia. Following the second dose, the patient began having a voiding problem and no organic pathology was found to explain the table of existing urinary retention [86].

Drug-induced UI in real clinical practice was also studied by the Hall et al. [87] in a population of 5503 men and women aged 30–79 living in Boston. They estimated the overall incidence of UI as 9.0% among women and 4.6% among men. Men and women with UI had a significantly older mean age compared to those without. Among women, the incidence was highest among users of certain antihistamines (28.4%) and angiotensin II receptor blockers (ARBs) (22.9%). Among men, the incidence was highest among ARBs (22.2%) and loop diuretic (19.1%) users [87].

Among cardiovascular drugs, the group most often reported in the literature in the context of inducing voiding disorders are calcium channel blockers. These drugs affect voiding by impairing the detrusor muscle to create enough contractile force. In a cross-sectional Elhebir et al. [88] study, assessing the occurrence of LUTS in the population of 278 medical inpatients (including 85 calcium channel blockers users) aged 72.1 ± 13.7 years, after adjusting for other risk factors and drugs it was found that patients on amlodipine/nifedipine and diltiazem/verapamil (compared to non-users) were more likely to suffer from LUTS. Patients on felodipine/lercanidipine were less likely to suffer from LUTS. Moreover, 22.4% of patients treated with calcium channel blockers were also on treatment for LUTS compared to 9.3% of the group that did not receive this group of drugs. Thus, this study confirmed the association between the use of calcium antagonists and the development of LUTS. Similarly, a systematic review by Salman et al. [89], in total analysing five relevant studies, found that three of them reported a significant relationship between calcium channel blocker use and LUTS development. In the one remaining study, they found that monotherapy of calcium channel blockers was linked to a higher prevalence of nocturia and voiding symptoms, but only in young females.

When considering drugs predisposing to urinary tract infections, the group most frequently described in clinical reports are sodium-glucose co-transporter 2 (SGLT2) inhibitors (gliflozins). In an observational Uitrakul et al. [90] study carried out in real clinical practice among patients with type 2 diabetes mellitus, it was revealed that the overall incidence rate of UTI was 33.49% in the SGLT2 inhibitor treated group vs. 11.72% in the group of patients without treatment with gliflozins. The incidence rates of UTI were not different between dapagliflozin and empagliflozin treatment (34.00% and 33.03%, respectively). A systematic review by Figueiredo et al. [91], based on an analysis of 23 clinical trials, also showed an increased incidence of UTIs in patients using gliflozins, both in patients on SGLT2i monotherapy or on combination therapy. In addition, the review pointed out that increased risk was predominantly associated with the use of dapagliflozin, canagliflozin, and tofogliflozin, regardless of the dosing. Dapafliflozin was also studied in a Khan et al. cross-sectional study [92] in 400 patients with diabetes receiving either 5 mg or 10 mg of dapagliflozin as an add-on therapy for the treatment of type 2 diabetes. The global prevalence of UTIs in diabetic patients treated with 5 mg or 10 mg of dapagliflozin was 5.3%. Women were more affected (76.2%) than men and UTIs were more prevalent in patients older than 50 years (85.7%) than in any other age group.

For anti-infective chemotherapeutics, a detailed study by Tasian et al. [93] showed an increased risk of developing kidney stones for specific groups of drugs. They analysed the health records of 13 million adults and children seen by general practitioners between 1994 and 2015. Prior antibiotic exposure was documented for nearly 26,000 patients with kidney stones, compared with nearly 260,000 control subjects. The study revealed that five of the classes were associated with a diagnosis of kidney stone disease in clinical practice: sulpha drugs, broad-spectrum penicillins, cephalosporins, fluoroquinolones, and nitrofurantoin. The authors also concluded that the use of these agents is associated with increased odds of nephrolithiasis, with the greatest odds for recent exposure and exposure at a younger age.

In the case of drug-induced ED, case studies are dominated by patients using drugs causing autonomic imbalance. Thus, drugs like beta-blockers (nonselective), alpha-blockers, anticholinergic drugs and other affecting the adrenergic and cholinergic system, along with other antihypertensive drugs, are most often cited as being responsible for ED. Similarly, clinical reports indicate the involvement of centrally acting drugs in the development of drug-induced ED. Clinical cases [94,95,96] disclosed in this review most often refer to drugs from the classes listed above.

The drug-related entities of retroperitoneal fibrosis were reported for hydralazine, hydrochlorothiazide and ampicillin therapy [97], as well as for pergolide administered in patients with Parkinson’s disease [98]. In the past, RPF has been reported as a consequence of methysergide therapy for headaches and migraines [99]. An increase in the risk of developing RPF has also been demonstrated for the new drug class used in the personalised therapy of lung cancer or melanoma-anti-programmed cell death 1 (anti-PD-1) antibodies [100].

In conclusion, the use of many drugs may contribute to the development of iatrogenic disorders of the kidneys and urinary tract. Pharmacotherapy implemented in a patient may cause various LUTS: UR or UI, UTIs, urolithiasis, ED or RPF. Among the drugs that may predispose to urological disorders, centrally acting drugs, antihypertensive agents, selected antibacterial chemotherapeutics, gliflozins and drugs affecting autonomic nervous system are most often mentioned in clinical reports.

## 8. Conclusions

The review of the literature indicates that adverse drug reactions manifesting themselves in the urinary tract are less frequently reported compared to, e.g., gastroenterological or dermatological drug side effects. The use of many drugs listed in this review may be also the potential cause of the increased risk of urological disorder development. Their diagnosis is difficult because it requires the exclusion of other potential organic and functional causes of the observed symptoms. Thus, knowledge of drug-induced urinary tract disorders is important for physicians who, in the course of diagnostic procedures, must remember to consider the possible drug-related background of the observed urological abnormalities, as well as for pharmacists providing a medication use review for the patient in terms of pharmacovigilance and recognising less common adverse drug reactions. Finally, the possibility of the drug-induced development of lower urinary tract disturbances is important for patients themselves, as these disorders reduce patients′ therapeutic adherence. Therefore, patients should be counselled to notify their healthcare provider if they notice urinary symptoms.

## Figures and Tables

**Table 1:** Drugs associated with an increased risk of urinary retention [18,27,28].

Drug Class		Examples	Postulated Mechanism Contributing to Urinary Retention
Drugs with anticholinergic effects–muscarinic receptor antagonists (MRA)	Tricyclic antidepressants	Amitriptyline, Imipramine, Clomipramine, Nortriptyline	Drugs with antimuscarinic effect can cause or exacerbate urinary retention due to the failure of cholinergic-mediated bladder contraction (especially in patients with a pre-existing bladder outlet obstruction, e.g., benign prostate hyperplasia).
	Classic antipsychotics	Thioridazine, Chlorpromazine, Chlorprothixene, Risperidone, Clozapine	
	H1-antihistamines (1st generation)	Chlorpheniramine, Triprolidine Cyproheptadine, Clemastine, Dimenhydrinate, Diphenhydramine, Doxylamine	
	Antispasmodics	Dicyclomine, Propantheline, Oxybutynin	
	Antiparkinsonian agents	Benzatropine, Orphenadrine, Procyclidine	
	Antiarrhythmic drugs class I	Disopyramide, Flecainide	
	Antimuscarinic bronchodilators	Ipratropium, Tiotropium, Glycopyrronium	
	Anticholinergic drugs used to treat overactive bladder	Oxybutynin, Fesoterodine, Solifenacin, Trospium, Tolterodine	
Premedication drugs (cholinolytics)		Atropine, Hyoscine	
Anaesthetics		Bupivacaine, Propofol, Ketamine, General anaesthetics of duration greater than 60 min	Inhibitory effects on the contraction of the bladder (inhibition of micturition reflex).
Opioids		Morphine, Pethidine, Pentazocine, Fentanyl	Urinary retention occurs due to the mu-receptor-mediated blockade of the sensory input from the bladder and the volume-evoked micturition reflex that results in decreasing the usual sensation of bladder fullness and bladder overdistention, and partially the anticholinergic effect of opioids. In addition, opioids can increase bladder sphincter tone due to excessive sympathetic stimulation, resulting in a bladder outlet restraint.
Non-steroidal anti-inflammatory drugs		Diclofenac, Ketoprofen	Impairment of the direct prostaglandin-induced bladder contractility and by the impairment of tachykinins stimulating neurokinin receptors on afferent nerves and detrusor smooth muscle.
Benzodiazepines		Diazepam, Clonazepam	Probably caused by detrusor muscle relaxation
Selective serotonin reuptake inhibitors (SSRI)		Fluoxetine, Sertraline, Citalopram	Serotonin is an important neurotransmitter which facilitates storage by increasing sympathetic activity and inhibiting parasympathetic control over voiding; thus, SSRI may cause an increase in the post-voiding residual volume (the volume of urine left in the bladder at the end of micturition).
Calcium channel blockers (CCB)		NDHP blockers: Diltiazem and Verapamil DHP blockers: Amlodipine and Nifedipine (the highly vascular selective DHP Felodipine and Lercanidipine seem to have less effect on micturition	Reduce bladder smooth muscle contractility via the inhibition of the calcium influx.
Alpha-1-agonists		Methoxamine, Phenylephrine, Ethylephrine	Binding to the alpha-1A/D adrenergic receptors of the internal urethral sphincter resulting in a higher tone of the internal sphincter.
Non-selective alpha-agonists		Pseudoephedrine (e.g., found in many over-the-counter cold remedies)	

**Table 2:** Drugs associated with an increased risk of urinary incontinence [27,28,32,33,35].

Drug Class	Examples	Postulated Mechanism Contributing to Urinary Incontinence
Antipsychotics	Chlorpromazine, Thioridazine, Chlorprothixene, Trifluoperazine, Fluphenazine, Haloperidol	Stress urinary incontinence: These drugs decrease urethral sphincter tone (e.g., via the inhibition of alpha-adrenoreceptors or as a result of a complex action on the central and peripheral serotonin or muscarinic or GABA receptors); thus, situations related to increased intra-abdominal pressure may result in a breakdown of the sphincter pressure and the possibility of urine leakage.
Alpha-adrenoreceptor antagonists	Prazosin, Doxazosin, Terazosin, Phenoxybenzamine, Phentolamine	
Benzodiazepines and non-benzodiazepine sedatives	Diazepam, Zopiclone, Zolpidem	
Other drugs	Reserpine, Misoprostol, Urapidil	
Parasympathomimetic (direct and indirect)	Acetylcholine esterase inhibitors	Urge urinary incontinence: These drugs may increase involuntary detrusor contractility during the storage phase of the bladder, resulting from the stimulation of muscarinic receptors or as a result of a complex action on other central and peripheral receptors (e.g., serotonin ones) or estrogen/progesterone receptors located in the urethra, vesical triangle, sacrouterine ligament, levator ani muscles and pubocervical fascia.
Antidepressants	Amitriptyline, Nortriptyline, Desimipramine	
Serotonin 5-HT4 receptor agonists	Cisapride, Tegaserod, Prucalopride	
Hormone replacement therapy	Oestrogen/Progesterone preparations	
Drugs listed in Table 1 (e.g., antimuscarinics, antihistamines, antiparkinsonian agents, beta-adrenergic agonists, calcium channels blockers, opioids, sedatives)		Overflow urinary incontinence: These drugs contribute to an inability to expel urine and consequently, a secondary increase in intravesical pressure, breaking the sphincter pressure and causing a leakage of urine. Angiotensin receptor blockade also decreases both detrusor contractility and urethral sphincter tone, leading to increased stress urinary incontinence, which may be worsened by a cough related to the use of these drugs.
Angiotensin-converting enzyme inhibitors/Angiotensin receptor antagonists	Benazepril, Captopril, Enalapril, Perindopril, Quinapril	
Miscellaneous	Alcohol, Caffeine, Diuretics	These compounds contribute to excessive urine production by inhibiting the reabsorption of electrolytes and water from primary urine or by enhancing glomerular filtration. Thus, they cause the bladder to fill faster and increase the frequency of urination.

**Table 3:** Drugs associated with an increased risk of urinary tract infection development [41].

Drug Class	Examples	Postulated Mechanism Contributing to Urinary Tract Inections
Gliflozins–inhibitors of kidney sodium-glucose transport proteins 2 (SGLT2)	Canagliflozin, Dapagliflozin, Empagliflozin	Gliflozins cause glycosuria, with subsequent bacterial colonisation of the urinary tract and genital area.
Immunosuppressive agents	Glucocorticoids, Azathioprine, Cyclosporin A, Sirolimus–rapamycin, Mycophenolate mofetil	These drugs cause the impairment of local and systemic immune mechanisms, thereby facilitating bacterial colonisation.
Drugs with anticholinergic effects listed in Table 1 (e.g., antimuscarinics, antihistamines, antiparkinsonian agents, beta-adrenergic agonists, calcium channels blockers, opioids, sedatives)		These drugs affect the micturition by inhibiting the contractile activity of the bladder, dependent on the activation of muscarinic receptors, resulting in urinary retention in the bladder, which facilitates the bacterial colonisation of the bladder and the reduced evacuation of bacteria along with the urine stream.
Benzodiazepines, Calcium channels antagonists, Opioids, and Non-steroidal anti-inflammatory drugs (NSAIDs) are listed in Table 2		These drugs may impair micturition through detrusor muscle relaxation and contribute to voiding reflex disturbances, either on a spinal or supraspinal level, resulting in reducing and delaying the emptying of the bladder, which provides better conditions for bacterial colonisation.
Miscellaneous	Antibacterial agents (Sulphonamides, Ciprofloxacin, Ampicillin), Antacids, Calcium, Vitamin D and/or C, Laxatives, Loop diuretics (Furosemide), Anhydrase inhibitors (Acetazolamide), Xanthine oxidase inhibitors (Allopurinol)	These drugs or their metabolites may precipitate in the urinary tract and/or these drugs change the pH of the urine, contributing to the crystallisation of endogenous lithogenic substances in the urinary tract, thereby enabling the formation of urinary stones.

**Table 4:** Drugs associated with an increased risk of kidney stones development [52,61,62,63].

Drug Class	Examples	Postulated Mechanism Contributing to Kidney Stone Development
Drug-containing stones		
Sulfonamides	Sulfadiazine, Sulfaguanidine, Sulfamethoxazole, Sulfasalazine	These drugs and their metabolites are poorly soluble in urine; in the case of long-term treatment they may precipitate in urine.
Aminopenicillins Cephalosporins	Ampicillin, Amoxicillin, Ceftriaxone	
Quinolones	Pipemidic acid, Ciprofloxacin, Norfloxacin	
Other antibacterial drugs	Nitrofurantoin	
Protease inhibitors	Indinavir, Nelfinavir, Atazanavir	
Antacids aluminum hydroxide	Magnesium trisilicate, Aluminum hydroxide	
Miscellaneous	Triamterene, Allopurinol, Ephedrine, Acyclovir, Methotrexate	
Drug-induced “metabolic stones”		
Calcium-containing supplements	Many preparations are commercially available, also as dietary supplements	These drugs enhance intestinal calcium absorption, leading to hypercalcemia and the hypercalciuric state.
Vitamin D-containing supplements		
Loop diuretics	Furosemide	
Anhydrase inhibitors	Acetazolamide, Zonisamide, Topiramate	These drugs inhibit bicarbonate reabsorption and hydrogen ion excretion in proximal tubules, leading to systemic metabolic acidosis, an increase in urinary pH and a decrease in urinary citrates.
Laxative drugs (when abused)	Hyperosmotic or stimulant agents	These drugs, when abused, cause increased gastrointestinal fluid and potassium loss and low urinary output. The potassium depletion contributes to intracellular acidosis, compensated by ammonia genesis enhancement in kidney proximal tubules and increased citrate reabsorption, potentiated by increased expression of the H+/K+ activity in the distal tubules.
Corticosteroids	Cortisol	These drugs promote the release of calcium from the bones and lead to the hypercalciuria and hyperphosphaturia state.
Ascorbic acid (vitamin C)	Many preparations are commercially available, also as dietary supplements	The excess of vitamin C is metabolised to form oxalates and it increases the urinary oxalates excretion. Moreover, high doses of vitamin C cause acidification of the urine.
Xanthine oxidase inhibitors	Allopurinol	The drug inhibits the biotransformation of hypoxanthine into xanthine and the final synthesis of uric acid, leading to the formation of xanthine-containing purine stones.
Uricosuric drugs	Benzbromarone, Probenecid	These drugs reduce hyperuricemia by enhancing urinary uric acid excretion, leading to the formation of uric acid-containing purine stones.

**Table 5:** Drugs that may cause erectile dysfunction [65,66,67].

Drug Class	Examples	Postulated Mechanism Contributing to Erectile Dysfunction
Beta-adrenergic antagonists	Non-selective (Propranolol, Sotalol, Oxyprenolol)	Beta-blockers suppress the sympathetic outflow and inhibit the beta-2-mediated peripheral relaxation, indirectly predisposing patients to the stimulation of alpha-adrenergic receptors that promote vasoconstriction. Non-selective beta-antagonists have a higher potential to cause ED resulting from the vasoconstriction within the corpora cavernosa.
	Beta1-selective agents (Metoprolol, Atenolol)	
Thiazide diuretics	Chlorthalidone Hydrochlorothiazide	Thiazides can cause sexual dysfunction through direct effects on vascular smooth muscles or by interfering with catecholamine responsiveness. Moreover, the thiazide-induced sodium loss and lower blood pressure may contribute to increased alpha-2 adrenergic activity, leading to decreased blood flow to the corpora cavernosa.
Potassium-sparing diuretics (aldosterone inhibitors)	Spironolactone, Eplerenone	These agents exhibit strong antiandrogenic effects by binding to mineralocorticoid receptors, causing gynecomastia, mastodynia and ED.
Statins	Simvastatin	The statin-induced decrease in cholesterol synthesis may negatively impact the male steroidal hormone level (androgens–testosterone). Moreover, statins inhibit not only HMG-CoA, but also 17-ketosteroid-oxidoreductase that catalyses the synthesis of androstenedione and testosterone. Thus, a decrease in serum testosterone level is observed in males with hypercholesterolemia treated with statins.
Tricyclic antidepressants	Imipramine, Clomipramine	The increased availability of serotonin due to the inhibition of its reuptake evoked by SSRI/SNRI leads to the increased binding of this neurotransmitter to the 5-HT2 and 5-HT3 receptors which disturbs sexual functions. Moreover, the complex, multi-receptor effect of tricyclic antidepressants also contributes to peripheral circulatory disturbances. In general, antidepressants with lower affinity to dopamine and noradrenaline receptors have a lower influence on ED.
Selective serotonin or noradrenalin reuptake inhibitors (SSRI/SNRI)	Sertraline, Citalopram, Paroxetine, Escitalopram, Duloxetine, Venlafaxine	
First generation antipsychotics	Haloperidol, Clozapine, Thioridazine	First-generation antipsychotics block central D2 receptors, decreasing the inhibition of prolactin production in the anterior pituitary gland, resulting in secondary disturbances in testosterone synthesis. Moreover, antipsychotics may also impair peripheral vasodilatation via cholinergic antagonism
Antiepileptics	Phenytoin, Phenobarbital, Carbamazepine	Both antiepileptic drugs and seizures interfere with the hypothalamic-pituitary-gonadal axis and alter sex steroid hormone production. Moreover, some antiepileptics induce hepatic enzymes resulting in increases of sex hormone binding globulin, which leads to lower free testosterone level.
Non-steroidal anti-inflammatory drugs	Naproxen, Diclofenac, Ibuprofen	Cyclooxygenase inhibition decreases the production of prostanoids (prostaglandins and thromboxanes) with a subsequent decrease of vasodilatory, and the erectile effect of nitric oxide (NO).
Muscle relaxants	Baclofen	The inhibition of afferent stimulation from the penis, somatic motor efferents mediating perineal muscle contraction and visceral motor efferents controlling the penile vasculature.
H2 receptor antagonists	Cimetidine, Ranitidine, Famotidine	H2 receptors are strongly associated with the relaxation of the corpus cavernosum; thus, H2 receptor antagonism results in decreased relaxation, increased contraction and a decreased potential for erection. Moreover, cimetidine exhibits anti-androgen, which affects and causes hyperprolactinemia
5-alpha reductase inhibitors	Finasteride, Dutasteride	These drugs inhibit type II 5-alpha reductase–the enzyme that catalyses the conversion of testosterone into more biologically potent dihydrotestosterone (DHT), with the subsequent decrease in nitric oxide synthase activity
Cancer drugs	Cisplatin, Vincristine, Vinblastine	Chemotherapy is known to cause ED by causing neural and vasculature damage. Moreover, long-acting gonadotrophin-releasing hormone agonists used for prostate and breast cancer cause hypogonadism, leading to a reduction in sexual desire and ED.
Miscellaneous	Antiandrogens (cyproterone), Digoxin, Steroids, Immunosuppressants (Sirolimus, Everolimus), Protease inhibitors in HIV	Various, complex mechanisms: the blocking of the androgen receptors, the lowering of serum testosterone and the impairment of gonadal functions.

**Table 6:** Drugs with suggested association with retroperitoneal fibrosis [27,28,71,72,73].

Drug Class	Examples	Postulated Mechanism Contributing to Retroperitoneal Fibrosis
Non-opioid and opioid analgetics	Aspirin, Paracetamol, Codeine	Drugs may initiate an immune response in the retroperitoneum followed by a progression to a chronic inflammatory process in the form of the proliferation of fibroblasts and the deposition of collagen, fibronectin, tenascin and glycosaminoglycans. The process of fibrosis involves the adjacent organs, including the ureters, causing their compression and possible symptoms of obstructive uropathy.
Antihypertensives	Hydralazine, Methyldopa, Reserpine, Hydrochlorothiazide	
Beta-adrenergic antagonists	Non-selective (Propranolol, Sotalol, Oxyprenolol)	
	Beta1-selective agents (Metoprolol, Atenolol)	
Cytostatics	Carboplatin, Methotrexate	
Biological drugs	Etanercept (TNF-alpha receptor blocker), Infliximab (anti-TNF-alpha monoclonal antibody)	
Miscellaneous	Egrot derivatives, Haloperidol, Ampicillin, Glibenclamide,	

## Data Availability

Data sharing is not applicable to this article.

## Abbreviations

ADR	adverse drug reaction
CC	chemical cystitis
CIOMS	Council for International Organizations of Medical Sciences
CNS	central nervous system
DU	detrusor underactivity
ED	erectile dysfunction
HC	hemorrhagic cystitis
KIC	ketamine-induced cystitis
LUTS	lower urinary tract symptoms
OAB	overactive bladder
PAG	periaqueductal gray
PMC	pontine micturition centre
RPF	retroperitoneal fibrosis
UAB	underactive bladder
UI	urinary incontinence
UR	urinary retention
UTI	urinary tract infection
WHO	World Health Organization
